# Warming temperature reduces the risk of pre-harvest freezing injury and modifies variety suitability in the main winegrape-growing regions of China

**DOI:** 10.1093/hr/uhaf176

**Published:** 2025-07-07

**Authors:** Huiqing Bai, Jianqiang He, Cornelis van Leeuwen, Rafiq Hamdi, Erna Blancquaert, Gregory V Jones, Zhanwu Dai

**Affiliations:** State Key Laboratory of Plant Diversity and Specialty Crops, Beijing Key Laboratory of Grape Sciences and Enology, Institute of Botany, Chinese Academy of Sciences, Beijing 100093, China; Institute of Environment and Sustainable Development in Agriculture, Chinese Academy of Agricultural Sciences, Beijing 100081, China; Key Laboratory for Agricultural Soil and Water Engineering in Arid Area of Ministry of Education, Northwest A&F University, Yangling 712100, Shaanxi, China; EGFV, Univ. Bordeaux, Bordeaux Sciences Agro, INRAE, ISVV, F-33882 Villenave d’Ornon, Talence, France; Royal Meteorological Institute of Belgium, Brussels, Belgium; South African Grape and Wine Research Institute (SAGWRI), Department of Viticulture and Oenology, Stellenbosch University, Private Bag X1, Matieland, 7600, South Africa; Department of Environmental Science, Policy, and Sustainability, Southern Oregon University, Ashland, OR 97520, USA; Abacela Vineyards and Winery, Roseburg, OR 97471, USA; State Key Laboratory of Plant Diversity and Specialty Crops, Beijing Key Laboratory of Grape Sciences and Enology, Institute of Botany, Chinese Academy of Sciences, Beijing 100093, China; China National Botanical Garden, Beijing 100093, China

## Abstract

Rising temperatures cause advanced phenology of grapevines and increased sugar concentration in berries, which ultimately modify variety suitability in a given region. Here, four bioclimatic indices and a refined grapevine sugar ripeness (GSR) model were employed to assess the suitability of six winegrape varieties across six winegrape-growing regions of China under historical climate conditions (1961–2020). First, four indices were compared between two periods, one before (P1) and one after (P2) an abrupt climate change events identified during 1988–2002 in these regions. Results showed three temperature- related indices increased in six regions, while the first fall frost day was delayed by 0–16 days in five out of the six regions during P2 compared with P1. Second, GSR model was applied to simulate target sugar concentrations as a proxy for grape harvest dates (GHDs). Direct utilization of original GSR model yielded unsatisfactory predictions with clear bias. Consequently, GSR model was recalibrated with local data to obtain an acceptable performance with *R*^2^ and NRMSE values of 0.83 and 2.8% as well as 0.83 and 3.1% for the calibration and validation datasets, respectively, and further simulated GHDs of six varieties with advanced values of 6–30 days in six regions for P2 in comparison with P1. To provide a holistic view of freezing injury risk before harvest, comprehensive freezing injury index (CFI) was developed by integrating the frequency, duration and severity of the freezing risk. CFI decreased (2–60%) during P2 in all regions and the magnitude of decrease was elevation dependent. These findings provide valuable insights into the selection of varieties that can more reliably achieve fully mature fruit, producing more balanced wines with greater typicity under warming climate.

## Introduction

Climate change is an ongoing concern with potential impacts on crop production [1, 2]. Grapevine is a perennial and highly climate-sensitive crop, making it vulnerable to climate change, especially to rising temperatures and increased variability [3–7]. Extensive research has confirmed that grapevines are influenced by microclimate, making them susceptible to the changes in temperature. These changes can significantly affect the phenology, yield and quality potential of winegrapes [8–11]. For example, advanced phenology due to rising temperatures may shift ripening period into the warmest months of the year (July or August, instead of September in the Northern Hemisphere), resulting in negative impacts on the balance between sugar, organic acids and aroma profiles [12–16]. Obviously, increasing temperatures pose a substantial threat to the production of high quality wines. Consequently, it is crucial to address the challenges posed by climate change and develop adaptation strategies to mitigate its impact on wine production. Understanding the relationship between climate variables and the timing of ripeness for winegrapes can help to minimize negative effects and optimize wine quality in a changing climate condition.

To investigate climate variations in winegrape-growing regions throughout history and future decades, researchers have utilized different bioclimatic indices as metrics to assess the suitability of these regions for wine production and explore potential geographical shifts in response to climate change. Commonly used indices include the Winkler index (WI) [17] which is based on growing degree days (GDD) [18], the Huglin index (HI) [19] and cool night index [20], etc. These indices have been widely employed to assess the suitability of winegrape-growing regions across different countries under present conditions and as the result of climate change [21–26]. Studies based on these indices have revealed that suitable viticulture areas are expected to expand towards higher elevations, higher latitudes and closer to the coast as the climate continues to warm. This shift in suitability may result in the changes of winegrape varieties and wine styles within specific regions as they adapt to a changing climate [27–30]. Bioclimatic indices can help researchers to gain insight into the potential impacts of climate change on viticulture. These findings can assist decision-makers in the implementation of adaptation strategies for the wine industry in the face of future climate challenges.

Several studies have emphasized the potential of the diversity of winegrape varieties as a lever for adaptation to climate change [31, 32]. Currently, over 6000 winegrape varieties of the *Vitis vinifera* species have been identified [33–35], but only 16 varieties cover half the world’s winegrape growing areas in 2016, indicating significant potential for enhancing variety improvement [36, 37]. The timing of major phenological stages (budbreak, flowering, véraison) depends on the variety (genetic component) and on the climate (in particular temperature, environmental component) [38]. Hence, the phenology of grapevine varieties is a sensitive biological indicator of climate change and an important criterion for evaluating variety suitability [28, 39]. The warming climate has already affected grapevine phenology and this trend is predicted to continue [40–42]. This advancement in phenology leads to advanced ripening and higher sugar concentrations at harvest, which translates into higher alcohol wines after fermentation [43–45]. This goes against a global trend of consumers are shifting to lower alcohol wines from a health and social perspective. Therefore, it is crucial to understand how temperature impacts the timing of phenological stages across different varieties for specific winegrape-growing regions.

Phenological models are useful tools for predicting grapevine development [46–48]. Several studies have assessed the impacts of climate change on the timing of phenological stages based on phenological models in different winegrape-growing regions of the world [49–51]. These models primarily use temperature as input data as well as employ a start date and temperature thresholds that have been tested for numerous varieties under various climatic conditions [37, 52, 53]. Among these models, the Grapevine Sugar Ripeness model (GSR; [53]), predicts the day of year (DOY) to reach given sugar concentrations for a wide range of grapevine varieties. This model is valuable for assessing variety suitability under climate change. Harvest dates were shown to be well predicted with the GSR model for Chardonnay, Cabernet-Sauvignon, Merlot, Pinot noir, Riesling and Syrah in Bordeaux, France; Champagne, France and Marlborough, New Zealand [54]. Modelled simulations of phenology were also acceptable in Mediterranean climate conditions, including GDD and a Sigmoid model for budbreak, flowering, véraison and harvest time [55, 56]. However, only few studies addressed the issue of assessing and predicting the phenology of winegrape varieties with specific models in the winegrape-growing regions of China, which has continental climate conditions, different from the climates used in developing the GSR model. Wang et al. evaluated the performance of numerous models (GDD, BRIN, Caffarra’s, Wang and Engel’s model) for three phenological periods (budbreak, flowering and véraison) and proved their accuracy in winegrape-growing regions of China [54]. Nevertheless, there is a lack of research on the simulation of grape harvest dates (GHDs), which is crucial for the assessment of cultivar suitability in a given region.

To obtain fully mature fruit at harvest is a prerequisite for the production of high-quality wine showing the unique characteristics of a particular variety. However, achieving optimal ripeness can be challenging in continental climates due to the threat of freezing injury before harvest, and may significantly impact variety suitability in specific regions [57]. In these conditions, variety choices as a function of local thermal conditions are of utmost importance to reach both yield and wine quality targets. Grapes need to reach full ripeness not too early in the season for optimal expression of the variety’s distinctive traits and qualities, but early enough so that the grapes reach full ripeness before the first frost event in the autumn. Currently, the low suitability between the ripeness of winegrapes and their respective growing regions directly affects the expression of fruit quality and serves as a concealed limiting factor for enhancing the quality of winemaking materials [58].

The northern winegrape regions of China are characterized by a typical continental monsoon climate, which necessitates the covering of most grapevines with soil during the winter to avoid frost damage to the perennial parts of the vine [59]. Like other major international winegrape-growing regions, Cabernet-Sauvignon covers over 50% of the total planted winegrape area in China [60], despite the large diversity in climate conditions. Hence, this raises the question whether Cabernet-Sauvignon is the most suitable variety for all these regions, or if other varieties may be a better fit to local climatic conditions and how varietal suitability evolves over time in a changing climate. Bioclimatic indices and the GSR model are appropriate tools to analyze the changes in phenology and to assess variety suitability under evolving climate conditions in China for ensuring a sustainable industry. This study focusses on the following aspects (i) analyze the trend of bioclimatic indices in different wine-growing regions of China over the recent-past period (1961–2020); (ii) explore the change of GHDs for different target sugar concentrations (iii) and assess the suitability of six winegrape varieties under conticlimate change conditions based on the GSR model and the first fall frost day (FFD).

## Results

### Climatic characteristics of each winegrape-growing region under climate change

**Figure 1:**
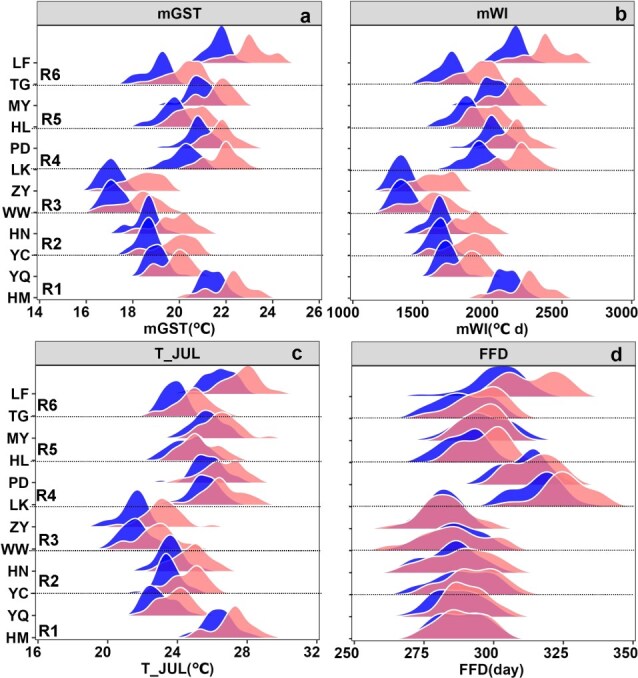
Ranges of average growing season mean temperature (mGST) **(a)**, W (mWI) **(b)**, average hottest month temperature (T_JUL) **(c)** and first fall frost day (FFD) **(d)** during 1961–2020 for 12 representative stations in China during P1 (before abrupt climate change) and P2 (after abrupt climate change). Note: HM, YQ, HN, YC, WW, ZY, LK, PD, HL, MY, TG and LF represent Hami, Yanqi, Huinong, Yinchuan, Wuwei, Zhangye, Longkou, Pingdu, Huailai, Miyun, Taigu and Linfen, respectively. R1, R2, R3, R4, R5 and R6 represent Xinjiang, Ningxia, Gansu, Shandong, Jing-Jin-Ji and Shanxi regions, respectively. Blue and red represent the period of P1 and P2, respectively.

Twelve representative stations all experienced abrupt climate change over the past 60 years (1961–2020) according to the Mann-Kendall trend test, and the abrupt years of each station ranged from 1988 to 2002 (Table S1). Then, the range and average values of four climatic characteristics as well as their change between P1 and P2 were analyzed in different winegrape-growing regions. The range and average values of mGST were 17.1–21.9°C and 19.5°C during P1. The increase of mGST ranged from 0.8 to 1.7°C across the studied regions, wherein the mGST increased more than 1.5°C in R2 (Ningxia) and R3 (Gansu) as well as above 1.0°C in R4 (Shandong) and R6 (Shanxi) during P2 (Fig. 1a). The mWI across the 12 representative stations showed a wide variation, ranging from 1344 to 2177°C·d during P1, and increased to 1601–2445°C·d during P2 (Fig. 1b). The trend of increased mWI was similar with the mGST in each region. The range and average values of T_JUL were 21.5–26.6°C and 24.3°C during P1. The T_JUL increased by 0.7°C to 1.9°C during P2 in comparison with P1. Among the locations, the increased T_JUL exceeded 1.7°C in R3 (Gansu) and 1.0°C in R2 (Ningxia) and R6 (Shanxi) during P2 (Fig. 1c). However, the FFD showed different patterns with the average values of 293 day (281–316 day) during P1 and 299 day (283–327 day) during P2. Its values slightly changed by 0.2 day and − 0.9 day, respectively, in Hami (HM) of R1 (Xinjiang) and Miyun (MY) of R5 (Jing-Jin-Ji), significantly advanced in Longkou (LK) of R4 (Shandong) and Linfen (LF) of R6 (Shanxi) with the values of 11 and 16 days, respectively, as well as 2–8 days in other regions during P2, respectively (Fig. 1d).

### Performances of the original and improved growing season ripening models

**Figure 2:**
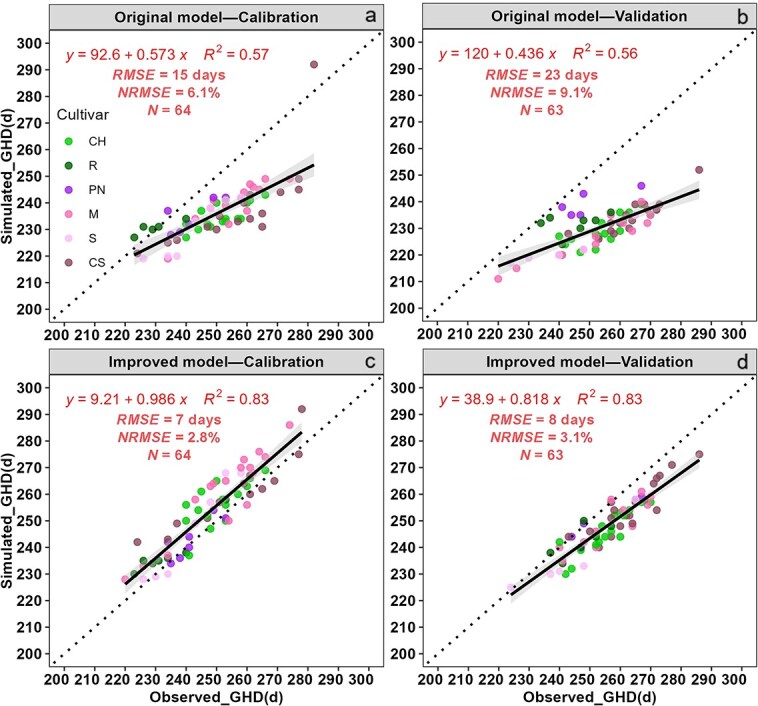
Comparisons of simulated and observed grape harvest dates (GHDs) for two white and four red varieties for original and improved GSR models. Dashed and solid lines represent 1:1 line and linear regression line, respectively. Note: CH, CS, M, PN, R and S represent Chardonnay, Cabernet-Sauvignon, Merlot, Pinot noir, Riesling and Syrah, respectively. *RMSE* and *NRMSE* represent the root mean-square errors and the normalized root mean-square errors between observed and simulated grape harvest dates, respectively. *N* represent the number of observed data.

The performance of the original and improved growing season ripening (GSR) models were evaluated by comparing simulated and observed GHDs for each variety under different sugar concentrations planted in 11 winegrape-growing stations (Table S2). The original GSR model was found to be inaccurate for winegrape-growing regions in China, as indicated by the clear bias, the high root mean-square errors (RMSE) of 15 days and 23 days, and the normalized root mean-square errors (NRMSE) of 6.1 and 9.1% for the calibrated and validated datasets, respectively (Fig. 2a and b). To address this issue, we further tuned the parameters of original GSR model (see details in the materials and methods section). The results indicated that the start dates when temperatures were higher than 10°C for each winegrape-growing region ranged from March 25 to May 3 in China (Table S3) and these values were used to set the *t*_0_ of the model. The parameters (*a* and *b*) related to the thermal requirements F^*^_s-target_ of each variety were re-estimated for the modified GSR model (Fig. 8). It turned out that a much higher value of thermal accumulation (F^*^_s-target_) was required for each of the six varieties to reach the same sugar ripeness in China than those in the original model that was calibrated and validated in Europe. After this re-parameterization, the RMSE of the improved model was 7 days for model calibration and 8 days for model validation (Fig. 2c and d). Moreover, the observed and simulated GHDs showed good agreement, with *R*^2^ values of 0.83 and 0.83 for the calibration and validation datasets, respectively. The improved model achieved reasonable simulation of GHDs, with NRMSE values of 2.8 and 3.1% for calibration and validation datasets, respectively. Generally, the improved GSR model, with adjusted parameters (Table 1), demonstrated the better performance in simulating the GHDs, even though it slightly underestimated the GHDs in the validation dataset (Fig. 2c and d).

**Table 1:** The modified parameters (*a* and *b*) of improved GSR model for six cultivars.

Cultivar	*a*	*b*	Cultivar	*a*	*b*
Chardonnay	7.34	1680	Merlot	7.05	1710
Riesling	11.02	1160	Syrah	7.02	1825
Pinot noir	5.36	1820	Cabernet-Sauvignon	8.04	1795

**Figure 3:**
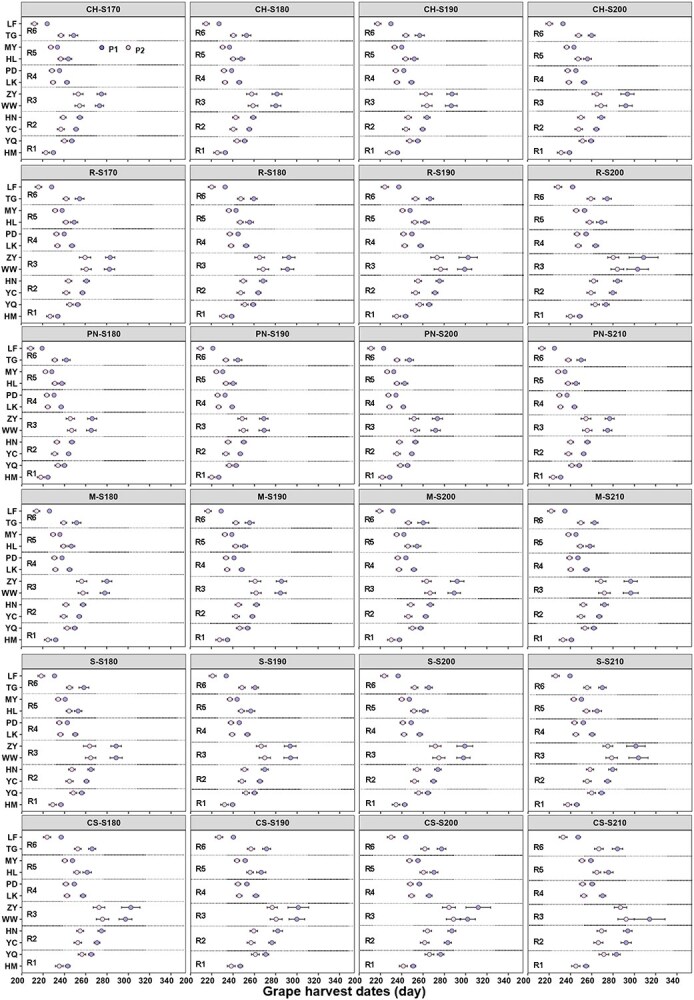
Predicted GHDs of six varieties under four target sugar concentrations during P1 (before abrupt climate change) and P2 (after abrupt climate change). Note: CH, CS, M, PN, R and S represent Chardonnay, Cabernet-Sauvignon, Merlot, Pinot noir, Riesling and Syrah, respectively. HM, YQ, HN, YC, WW, ZY, LK, PD, HL, MY, TG and LF represent Hami, Yanqi, Huinong, Yinchuan, Wuwei, Zhangye, Longkou, Pingdu, Huailai, Miyun, Taigu and Linfen, respectively. R1, R2, R3, R4, R5 and R6 represent Xinjiang, Ningxia, Gansu, Shandong, Jing-Jin-Ji and Shanxi regions, respectively. S170, S180, S190, S200 and S210 represent the sugar concentration of 170, 180, 190, 200 and 210 g/L, respectively.

### Change of GHDs under climate change

Figure 3 illustrated the changes of predicted GHDs from the improved GSR model for six varieties and four target sugar concentrations in six regions before (P1) and after (P2) abrupt climate change event. The range and average of DOY for GHDs during 1961–2020 were 207–307 and 245 days, 222–335 and 259 days, 229–340 and 276 days, 211–287 and 243 days, 212–309 and 245 days, 198–316 and 241 days, as well as their change trends ranged from 2–3, 2–6, 4–12, 2–4, 2–3 and 3–5 days/10y, respectively, in R1, R2, R3, R4, R5 and R6. The GHDs during P2 for the six varieties were all advanced in comparison with those of P1, and the advancements ranged from 6 to 29 days depending on the region, variety and sugar concentration at harvest. Among the six regions, the change of GHDs was the largest (19–29 days) in R3 (Gansu). Among the six varieties, the change of GHDs was the largest for Cabernet-Sauvignon (7–26 days) and the smallest for Pinot noir (6–16 days). For the other two red varieties, the changes of GHDs were 6–20 days for Merlot and 7–23 days for Syrah. For the two white varieties, the changes of GHDs were 6–19 days for Chardonnay and 7–23 days for Riesling. The impact contributions of region, variety and sugar concentration on the change of GHDs were determined through ANOVA analysis, which were 90.8, 3.4 and 1.3%, respectively. In addition, analyzing simulated GHDs of six varieties for each target sugar concentration over the past 60 years, it was found that some varieties were unable to reach the targeted sugar level in 2.8–100% of the years in Huinong of R2, Huailai of R5, Taigu of R6 and R3 (Gansu) during P1 (Table S4), with thermal accumulation calculating continuously from *t*_0_ to November 30th. For instance, when planting the late-ripening variety Cabernet-Sauvignon in Zhangye of R3, it could not reach the targeted sugar ripeness in 48.9% of years with a target sugar of 180 g/L and 100% of the years with a target sugar concentration of 210g/L during P1 (Table S5). This situation was mostly improved during P2, with assured full maturity in all years at 180 g/L sugar but still showing 17% of the years not reaching maturity at 210g/L sugar (Table S5). In contrast, the probability of not reaching full maturity for the early-maturing variety Pinot noir was the smallest (2.8–5.7%) during P1. Generally, as 80% of the years reach the required temperature for ripening, each variety was all suitable for planting both during P1 and P2 in Huinong of R2, Huailai of R5 and Taigu of R6. However, it was also found that the late-ripening variety (Cabernet-Sauvignon) was unsuitable for planting in R3 (Gansu) during P1, while the early-maturing varieties (such as Chardonnay and Pinot noir) was considered to be suitable for planting in these regions. Additionally, other varieties were also unsuitable for planting under high target sugar concentration in R3 (Gansu). Finally, the probability of not reaching target sugar concentrations was below 20% for all varieties during P2 (Table S4).

### Decreased freezing injury under climate change

**Figure 4:**
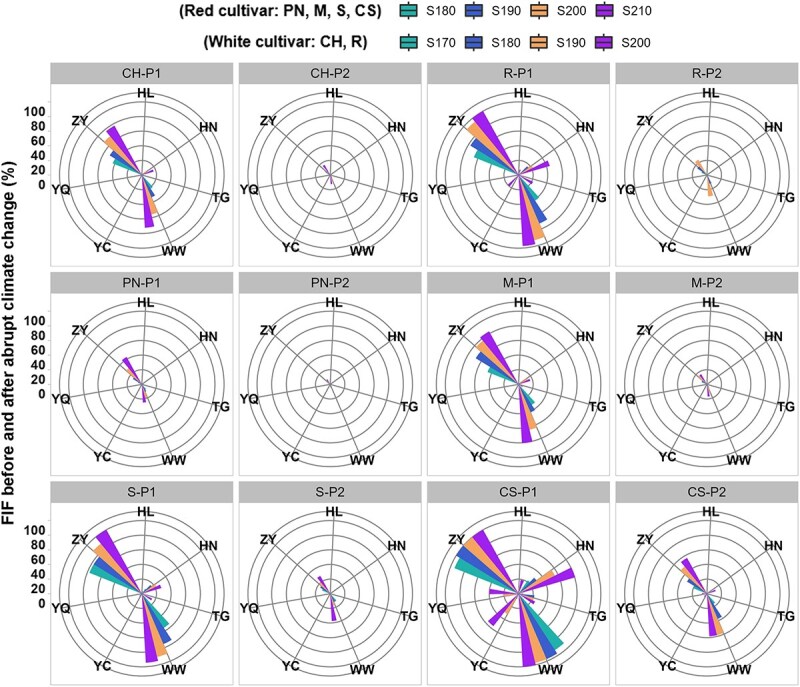
Freezing injury frequency (FIF) before harvest for four sugar concentrations during P1 (before abrupt climate change) and P2 (after abrupt climate change). Note: CH, CS, M, PN, R and S represent Chardonnay, Cabernet-Sauvignon, Merlot, Pinot noir, Riesling and Syrah, respectively. HL, HN, TG, WW, YC, YQ and ZY represent Huailai, Huinong, Taigu, Wuwei, Yinchuan, Yanqi and Zhangye, respectively.

Freezing injury occurred before harvest in five of the six regions studied, for different varieties, namely in Yanqi of R1, Ningxia of R2, R3 (Gansu), Huailai of R5 and Taigu of R6. The freezing injury frequency (FIF) is shown for six varieties before (P1) and after (P2) abrupt change in climate (Fig. 4). For each variety, the FIF generally declined by 2.4–75% for four sugar concentrations during P2. Among the varieties, Cabernet-Sauvignon suffered the most serious freezing injury before harvest in P1, while Pinot noir experienced the lowest FIF. The decrease in FIF from P1 to P2 was most dramatic for Cabernet-Sauvignon, with values of 3.9–19.2% in Huailai of R5, 6.3–21.4% in Taigu of R6, 3.3–33.3% in Yanqi of R1, 2.4–68.1% in Ningxia of R2 and 38.9–75% in R3 (Gansu) for four sugar concentrations. Pinot noir showed the lowest decrease in FIF, with values of 3.1% in Taigu of R6 and 5.7–34.9% in R3 (Gansu) for four sugar concentrations. For Chardonnay and Merlot, the decrease in FIF was below 20% in Huinong of R1 and Taigu of R6, while ranging from 18.1–66.9% in R3 (Gansu) for the four sugar concentrations considered after abrupt climate change. The changes in FIF were all below 20% in Yanqi of R1, Yinchuan of R2, Huailai of R5 and Taigu of R6, ranged from 2.8–44.4% in Huinong of R2 and 37.5–72.2% in R3 (Gansu) for Riesling and Syrah. Generally, the region with the most serious freezing injury was in R3 (Gansu). Higher risk of freezing injury existed under higher targeted sugar concentrations.

**Figure 5:**
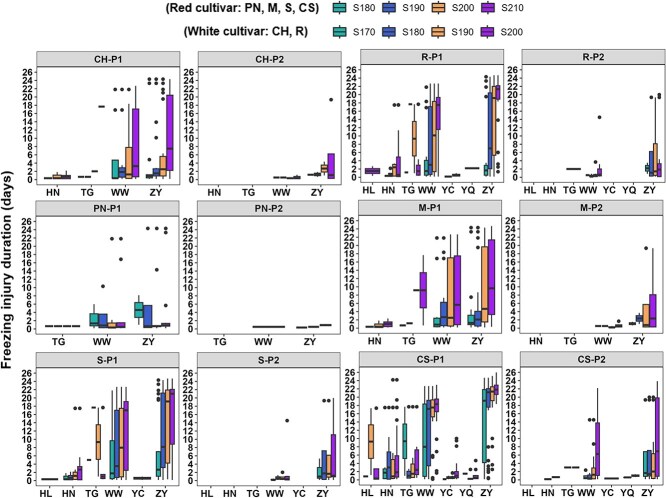
Freezing injury duration before harvest for four sugar concentrations during P1 (before abrupt climate change) and P2 (after abrupt climate change). Note: CH, CS, M, PN, R and S represent Chardonnay, Cabernet-Sauvignon, Merlot, Pinot noir, Riesling and Syrah, respectively. HL, HN, TG, WW, YC, YQ and ZY represent Huailai, Huinong, Taigu, Wuwei, Yinchuan,Yanqi and Zhangye, respectively.

The freezing injury duration (FID) of each variety was explored for four sugar concentrations before and after abrupt climate change (Fig. 5). During the P1 period, freezing injury occurred in Huinong of R2, R3 (Gansu) and Taigu of R6 for Chardonnay and Merlot, in Yanqi of R1, Huinong of R2, R3 (Gansu), Huailai of R5 and Taigu of R6 for Riesling and Cabernet-Sauvignon, in R3 (Gansu) and Taigu of R6 for Pinot noir, as well as in Huinong of R2, R3 (Gansu), Huailai of R5 and Taigu of R6 for Syrah. The FID ranged from 0 to 26 days during P1 while the FID decreased to below 20 days during P2 for each variety and for four sugar concentrations. When grapevines were planted in R3 (Gansu) with mGST values of <18°C, the FID was the largest for each variety with the values of 0–26 days during P1. The GST in Ningxia of R2 was <19°C. Their FID ranged from 0–2 days for Chardonnay and Merlot, 0–18 days for Riesling and Syrah and 0–24 days for Cabernet-Sauvignon in Huinong of R2 as well as 0–1 day for Riesling and Syrah and 0–10 days for Cabernet-Sauvignon in Yinchuan of R2. In Yanqi of R1, Huailai of R5 and Taigu of R6 with GST value of <20°C, the FID was 0–3 days in Huailai and 2 days in Yanqi for Riesling, 0–17 days in Huailai and 0–5 days in Yanqi for Cabernet-Sauvignon. In addition, the FID varied from 0–18 days for each variety in Taigu of R6. During the P2 period, freezing injury only happened in R3 (Gansu) for Chardonnay, Pinot noir, Merlot and Syrah. Generally, the maximum FID decreased by more than 15 days for Chardonnay and Riesling, while it decreased by more than 10 days for Cabernet-Sauvignon in R3 (Gansu).

**Figure 6:**
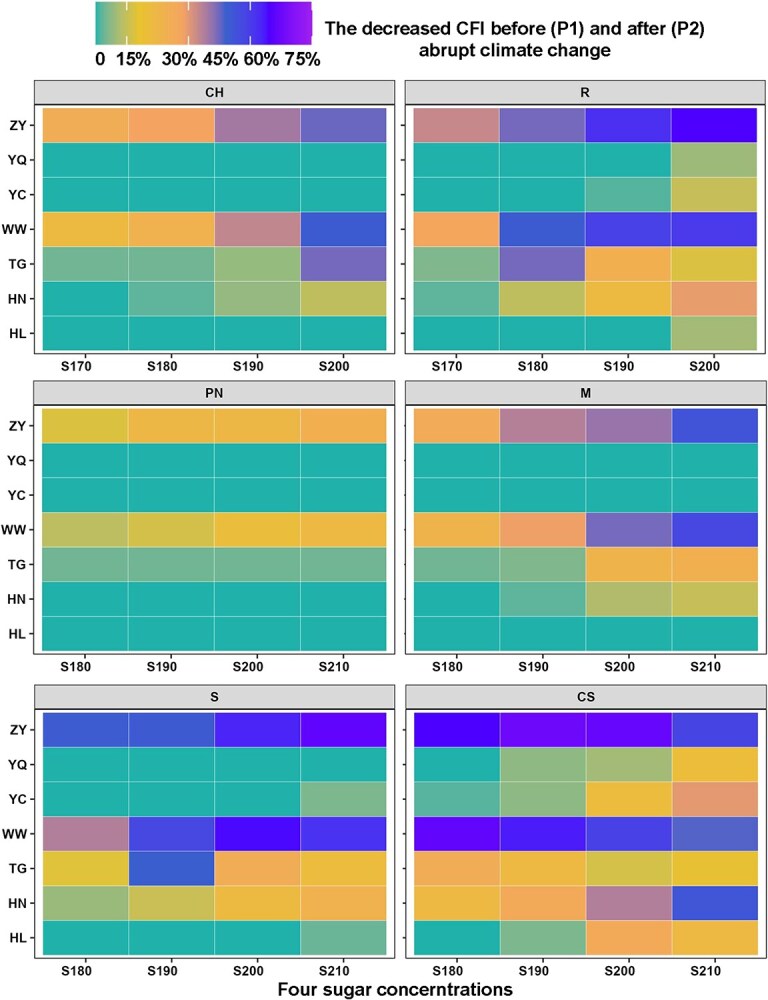
Decreased comprehensive freezing injury (CFI) for each variety under four sugar concentrations from P1 (before abrupt climate change) to P2 (after abrupt climate change). Note: CH, CS, M, PN, R and S represent Chardonnay, Cabernet-Sauvignon, Merlot, Pinot noir, Riesling and Syrah, respectively. HL, HN, TG, WW, YC, YQ and ZY represent Huailai, Huinong, Taigu, Wuwei, Yinchuan,Yanqi and Zhangye, respectively. S170, S180, S190, S200 and S210 represent the sugar concentration of 170, 180, 190, 200 and 210 g/L, respectively.

**Figure 7:**
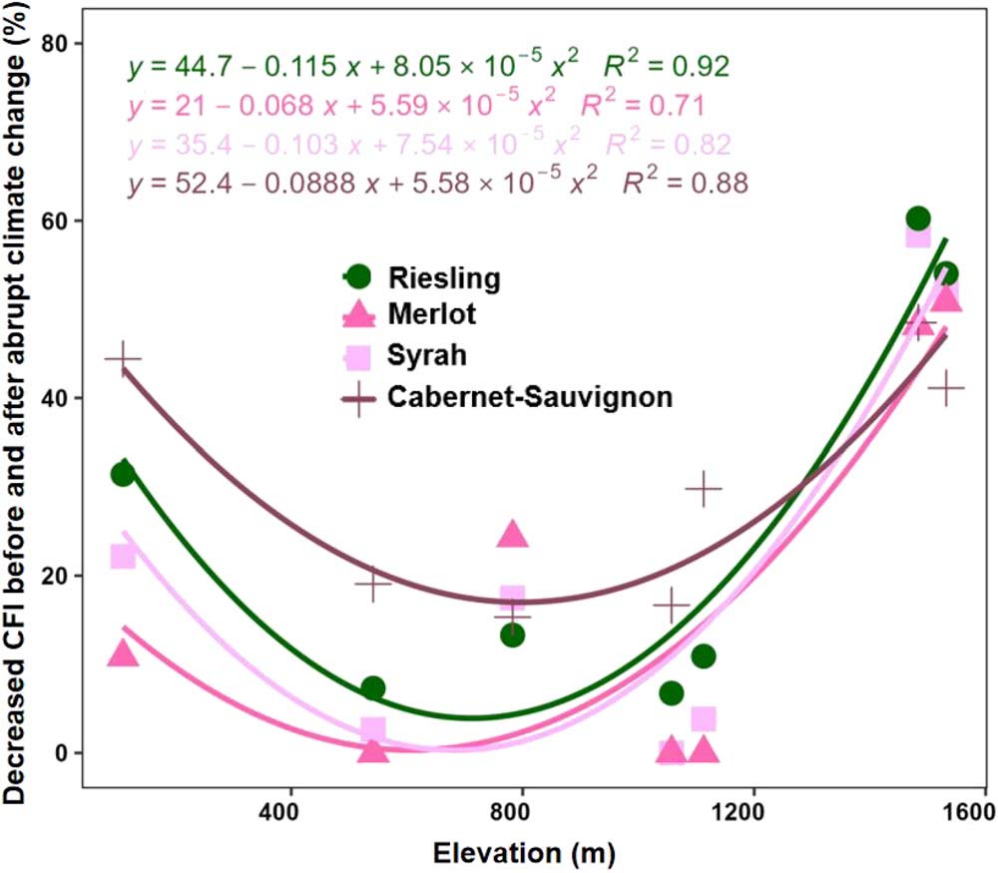
Relationship between elevation and decreased comprehensive freezing injury (CFI).

To provide a holistic view of freezing injury, a comprehensive freezing injury index (CFI) was developed by integrating the frequency, duration and severity of the freezing risk. The changes in CFI were shown in Fig. 6 for the six varieties before and after abrupt climate change in different winegrape-growing regions. The values of CFI decreased most significantly in R3 (Gansu), varying from 20–46% for Chardonnay, 29–60% for Riesling, 10–25% for Pinot noir, 23–51% for Merlot, 35–58% for Syrah and 41–60% for Cabernet-Sauvignon. For mid to late maturing varieties (e.g. Syrah, Riesling and Cabernet-Sauvignon), the CFI decreased by 0–44% in R2 (Ningxia). Moreover, the CFI decreased by 3–25% in Huailai of R5 for most varieties (except two early-ripening varieties), the CFI decreased by of 3–42% in Taigu of R6 for five of the six varieties (except Pinot noir). In addition, four varieties with obvious freezing injury were selected to explore the relationship between elevation and the decreased CFI. The decreased CFI exhibited an initial decline and then followed a subsequent increase with the ascending elevation, and the *R*^2^ values for each variety ranged from 0.71 to 0.92 (Fig. 7).

## Discussion

### Impact of climate change on viniculture

Application of bioclimatic indices and GSR model enabled us to assess the probability of freezing injury before harvest and to recommend suitable varieties for the main winegrape-growing regions of China, both before and after an abrupt climate change during 1961–2020. Bioclimatic indices, such as the WI [18], HI [20], growing season mean temperature [60] and average hottest month temperature [22], have been utilized by researchers worldwide to characterize the climatic conditions in vineyard. In this study, four bioclimatic indices were selected (namely, the mWI, mGST, T_JUL and FFD) to analyze the climatic characteristics before and after the abrupt climate change in main winegrape-growing regions of China. The mWI and mGST represent heat requirements heat for grape growing, T_JUL is considered as an important factor influencing grape quality potential, and FFD indicates the number of days required for growth and development without freezing injury before harvest [61]. Our analysis revealed that the mGST increased by above 1.5°C in R2 (Ningxia) and R3 (Gansu) and above 1.0°C in R4 (Shandong) and R6 (Shanxi) during P2. Moreover, both R2 (Ningxia) and R3 (Gansu) belong to warm conditions while both R4 (Shandong) and R6 (Shanxi) changed from warm to hot conditions before and after abrupt climate change based on the metrics of Jones et al. [40]. In addition, the change trend of increased mWI was similar with the GST in each region. Among each region, T_JUL increased by 1.7°C in R3 (Gansu), which is consistent with the increased mGST. However, FFD showed different patterns and the advanced FFD was the lowest in R3 (Gansu) with the value of only 2 days. There was a significant advancement of FFD by 11 days and 16 days, respectively, in Longkou of R4 and Linfen of R6 after abrupt climate change, implying that these regions are more suitable for late-ripening varieties. These findings are consistent with the results reported by Wang et al. [62], who observed a significant delay of FFD in different regions of China under climate change. Overall, our study highlights the importance of utilizing bioclimatic indices to assess the climatic characteristics of winegrape-growing regions.

### Impacts of climate change on GHDs

Since there were noticeable changes in each bioclimatic index under abrupt climate change in the main winegrape-growing regions of China, the GSR model [53] was employed to assess the changes of GHDs for six varieties in each region. While the GSR model has been used to predict Pinot noir suitability in America [63] and as a decision support tool for winemaking in Portugal [64], the current study represents the first assessment of the grapevine sugar ripeness (GSR) model in China. The model was calibrated and validated with observed GHDs and sugar concentration of six varieties in 11 grape growing sites of China. Since simulated results of the original GSR model had large bias in days it was considered not satisfactory, and the model parameters were adjusted. It should be noted that the GSR model was developed, parameterized and validated based on actual GHDs and sugar concentrations from Europe, where the temperature after the April 1st is consistently above 10°C (Fig. S1). Therefore, the date when the temperature consistently exceeded 10°C was based on a five-day moving average and set as the starting date (*t*_0_) in the improved GSR model for conditions in China. Moreover, the model parameters related to the thermal requirements (F*) for each variety were reparametrized accordingly. The performance metrics (RMSE and NRMSE) of the modified GSR model indicated much better performance with the values of below 8 days and 3.2%, respectively. Furthermore, the modified GSR model was applied to simulate GHDs for the before mentioned six varieties, and it was found that the GHDs were advanced by 6–30 days in each region. The change of GHDs was the largest in R3 (Gansu) for four sugar concentrations after abrupt climate change, which is consistent with the change trend of mGST. Overall, the advanced GHDs in regions with lower temperatures were greater than in regions with higher temperatures. Additionally, late-ripening varieties exhibited a greater advance of GHDs compared to early-ripening varieties. Previous studies by Parker et al. [54] and Skahill et al. [63] also indicated that the GHDs have advanced for reaching specific target sugar concentrations under a warming climate. Parker et al. [54] demonstrated an advancement of GHDs of 7–10 days to reach a sugar concentration of 190 g/L based on the GSR model, while Skahill et al. [63] used the GSR model to simulate GHDs for Pinot noir at a sugar concentration of 220 g/L and observed advanced trends of phenological periods from the 1950s to the 2090s. These findings align with the results of this study. In summary, the warming climate will increase the probability of providing sufficient heat for grape maturation to reach higher sugar concentrations earlier in the season.

### Impacts of climate change on suitability of grape variety

This study aimed to determine the suitability of different winegrape varieties in various regions of China before and after abrupt climate change during 1998–2002 by combining the risk of freezing injury before harvest with the modified GSR model. The results revealed a decrease of FFI for the six varieties following the abrupt climate change. According to the findings of this study, the late-ripening varieties (i.e. Cabernet-Sauvignon and Riesling) and medium-ripening variety (i.e. Syrah) are recommended for cultivation in Huinong of R2 and Taigu of R6. It is noteworthy that Cabernet-Sauvignon (known for its late-ripening) can also mature adequately in Hami of R1 and Yinchuan of R2. For Chardonnay, Pinot noir and Merlot, the thermal conditions for successful ripening under low sugar concentration, can usually be achieved in R3 (Gansu). Additionally, in 80% of years, these varieties achieve sufficient ripeness to mitigate the risk of frost injury. Our conclusions also highlight a reduction in FID following abrupt climate change. Previous studies have indicated a shift in climatic conditions in grape-growing regions of China from the south to the north, along with changes in variety suitability based on relevant climatic indices [62, 63]. Moreover, regions such as Xinjiang, Northeast China, Ningxia, Shandong, Gansu and others are expected to expand their winegrape-growing areas and introduce late-ripening varieties due to the rising temperatures [64]. These findings provide valuable insights for better planning and selection of winegrape varieties in the context of climate change in China, improving the assessment only based on single climatic indices alone. Future research should take into account other phenological periods, including the dates of budbreak, flowering and véraison, meanwhile, the spring frost injury during budbreak, high temperature during flowering, véraison and maturity and other climatic indices affecting grape yield and quality need to be analyzed in depth under continuing climate change. Furthermore, expanding the evaluation of winegrape variety suitability to a regional scale should be undertaken to fine-tune assessment of variety suitability in China. These additional considerations would provide valuable insights when assessing the adaptability of grape varieties, even if the GHD is included.

## Materials and methods

### Study region and meteorological data

Winegrapes are mainly planted in multiple provinces, cities and autonomous in Northwestern and Northern China, among which Xinjiang, Ningxia, Shandong, Gansu, Jing-Jin-Ji and Shanxi account for more than 80% of the total planting area and production in the country (Fig. S1a). Each region has its unique terroir characteristics and climatic suitability for diverse varieties of winegrapes. In this study, two representative sites were selected for each region, and a total of 12 representative meteorological stations were chosen from the main winegrape-growing regions of China, namely Xinjiang (R1), Ningxia (R2), Gansu (R3), Shandong (R4), Jing-Jin-Ji (R5) and Shanxi (R6) regions (Table 2). For each winegrape-growing region, daily meteorological data of 1961–2020 and geographical information were obtained from China Meteorological Administration, including daily maximum and minimum temperatures (°C), daily precipitation (mm), longitude, latitude and elevation (m). Across the 12 selected representative stations, the annual average, maximum and minimum temperatures ranged from 6.6 to 15.9°C, 13.6 to 21.3°C and −0.9 to 10.9°C, respectively (Fig. S1b–d).

**Table 2:** Geographical information of the 12 representative stations in main wine producing regions of China.

Region/Municipality	Station	Latitude (^o^)	Longitude (^o^)	Elevation (m)
Xinjiang (R1)	Hami	42.81	93.52	738
	Yanqi	42.08	86.57	1057
Ningxia (R2)	Huinong	39.22	106.77	109
	Yinchuan	38.48	106.22	1113
Gansu (R3)	Wuwei	37.92	102.67	1532
	Zhangye	38.93	100.43	1484
Shandong (R4)	Longkou	37.62	120.32	5
	Pingdu	36.77	119.93	49
Jing-Jin-Ji (R5)	Huailai	40.40	115.5	542
	Miyun	40.38	116.87	73
Shanxi (R6)	Linfen	36.07	111.50	450
	Taigu	37.43	112.53	783

### Climatic indicators

A comprehensive set of key indicators allow assessing climatic suitability and risk in viticultural areas around the world [63, 65, 66]. To capture the climatic characteristics of main winegrape-growing regions in China, several commonly used climatic indicators were selected, including average GST, WI, average hottest month temperature (T_JUL) and FFD. The first three indicators (GST, WI, T_JUL) were shown to provide a comprehensive description of the viticultural climate across diverse winegrape-growing regions [17, 19, 60, 67]. The indicator of FFD (DOY) was unique to regions that need to consider this climatic variable as a limiting factor. Although Jones et al. [40] defined the growing season of grapevine ranged from April 1st to October 31st in the Northern Hemisphere, the actual growing season of grapevine is from April 1st to September 30th in main winegrape-growing regions of China. Therefore, both GST and WI were redefined by Wang et al. [63] to describe the climatic characteristics of Chinese winegrape-growing regions. Here, the growing season of grapevine was set as April 1st to September 30th to calculate the modified GST (mGST) and WI (mWI).


(1) \begin{equation*} mGST=\frac{\sum \limits_{Apr1}^{Sep30}\frac{T_{\mathrm{max}}+{T}_{\mathrm{min}}}{2}}{n} \end{equation*}

(2) \begin{equation*} mWI=\sum \limits_{Apr1}^{Sep30}\left(\frac{T_{\mathrm{max}}+{T}_{\mathrm{min}}}{2}-{T}_{base}\right) \end{equation*}

(3) \begin{equation*} T\_ JUL=\frac{\sum \limits_{Jul1}^{Jul31}\frac{T_{\mathrm{max}}+{T}_{\mathrm{min}}}{2}}{n} \end{equation*}

where *T*_max_ and *T*_min_ represent the daily maximum and minimum temperature, respectively. *Apr1*, *Jul1*, *Jul31* and *Sep30* represent April 1st, July 1st, July 31st and September 30th, respectively. *T*_base_ means the temperature threshold with the value of 10°C.

### Mann-Kendall trend test

The Mann-Kendall trend test [68, 69] was commonly used to test the significance of trends in the hydrometeorological time series [70, 71]. Here, the Mann-Kendall test (Eqs 4–7) was used to analyze the time series of annual average temperature in order to identify the year of abrupt climate change at each representative station of main winegrape-growing regions.


(4) \begin{equation*} S=\sum \limits_{i=1}^{n-1}\sum \limits_{j=1}^{n-1}\operatorname{sgn}\left({x}_j-{x}_i\right) \end{equation*}

(5) \begin{equation*} \operatorname{sgn}\left({x}_j-{x}_i\right)=\left\{\begin{array}{@{}ccc}+1& {x}_j-{x}_i>0& \\{}0& {x}_j-{x}_i=0& \\{}-1& {x}_j-{x}_i<0& \end{array}\right. \end{equation*}

(6) \begin{equation*} Z=\left\{\begin{array}{@{}ccc}\frac{S-1}{\sqrt{Var(S)}}& \left(S>0\right)& \\{}0& \left(S=0\right)& \\{}\frac{S=1}{\sqrt{Var(S)}}& \left(S<0\right)& \end{array}\right. \end{equation*}

(7) \begin{equation*} Var(S)=\frac{n\left(n-1\right)\left(2n+5\right)-\sum \limits_{m=1}^n{t}_m\left(m-1\right)\left(2m+5\right)}{18} \end{equation*}

where *n* represents the time series with the value of 60; *x_i_* and *x_j_* represent the annual average temperature in *i and j* year, *j* equals *i* plus 1; and *S* approximately obeys a normal distribution with the mean value of 0. *Z* value was applied to determine whether the time series data showed a significant trend; *Var(S)* represents the variance of the statistic *S*. *t_m_* represents the value corresponding to the number of *m*; and *Z* > 0 and *Z* < 0 represent an increasing and decreasing trend, respectively.

### Observations of GHD and sugar concentration

The main grape varieties planted for winemaking in China are predominantly red grapes, accounting for approximately 79% of the total surface area in production, while white grape cultivars make up the rest 20% in China (http://www.nxputao.org.cn/). Cabernet-Sauvignon, with an overall cultivation area of 23 000 hectares, has become the most widely planted international variety in China. Other international red varieties include Merlot, Cabernet franc, Syrah, Pinot noir and etc. And LongYan, Chardonnay, White Riesling are the common white varieties (http://www.nxputao.org.cn/). In this study, two white varieties (Chardonnay and Riesling) and four red varieties (Pinot noir, Merlot, Syrah and Cabernet-Sauvignon) were selected to assess their variety suitability in main winegrape-growing regions of China. Observed GHDs (*n* = 127) and corresponding sugar concentrations for these six varieties were collected for the period 1994 to 2018 at eleven sites from the CNKI website (https://www.cnki.net/) in order to calibrate the parameters of the phenological model (Table S2). Half of the observation data (across all varieties) were used to fit the most accurate model, while the rest half (across all varieties) were implemented for model validation.

**Figure 8:**
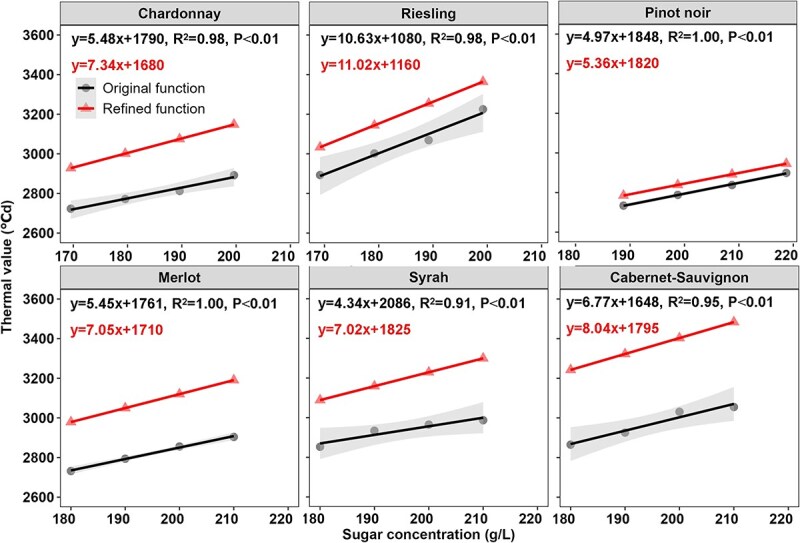
Linear regressions fitted using the original GSR parameters and re-fitted parameters *a* and *b* for Chinese climatic conditions. *y* and *x* represent the thermal value (F^*^_s-target_) and targeted sugar concentrations (S-target), respectively. Filled circles and their corresponding line are the actual values and regression line from the original model [48], the shading represents the 95% confidential intervals. Solid triangles and their correspondingline are the simulated values and fitted line after tuning parameters of model to Chinese climatic conditions.

### GSR model for simulating GHDs

The GSR developed by Parker et al. [48] was applied to simulate GHDss under target sugar ripeness (170, 180, 190, 200 and 210 g/L) for the two white cultivars (i.e. Chardonnay and Riesling) and four red cultivars (i.e. Pinot noir, Merlot, Syrah and Cabernet-Sauvignon). The GSR model is a linear model with three parameters of *T_b_*, *t*_0_ and F^*^_s-target_*.*


(8) \begin{equation*} \sum \limits_{t_0}^{t_{GHD}}{T}_d-{T}_b={F}^{\ast }_{S-targ et} \end{equation*}

where *T_d_* denotes daily average temperature greater than *T_b_*; *T_b_* represents the base temperature above which temperature summations starts; *t*_0_ is the date when temperature summations starts, *t*_GHD_ is the date of sugar ripeness; and F^*^_s-target_ is the thermal value when grape berry reaches a given sugar ripeness (i.e. target sugar concentration). In the original GSR model, *T_b_* and *t*_0_ are fixed as 0°C and 91 d (or April 1st), regardless of grape variety. In contrast, the thermal value (F^*^_s-target_) is variety specific and depends on the target sugar concentration in the berries of each variety [48] (Table S3).

Since a direct application of the original GSR model did not yield satisfactory reproduction of the observed harvest dates in this study (see details in results), the model was re-parameterized. In fact, the original model was developed based on the observations from Europe [48], which has Mediterranean and Atlantic climate conditions, meaning that the daily average temperature after the April 1st start date (i.e. *t*_0_ = 91) is mostly above 10°C (Fig. S2). Climate conditions are very different in the main winegrape-growing regions of China (Table S4). To take into account this kind of difference, the parameter *t*_0_ was re-estimated as the date when daily mean temperature consistently surpassed 10°C, through a five-day moving average. Additionally, the thermal value (F^*^_s-target_) required to meet the maturity of grapevines was provided for different target sugar concentrations*.* Therefore, the values (F^*^_s-target_) for different targeted sugar concentrations at harvest ([S*-*target]) could be described with a linear function (Eq. 9).


(9) \begin{equation*} {F}^{\ast }_{S-targ et}=a\times \left[S-target\right]+b \end{equation*}

where [S-target] is the targeted sugar concentration at harvest; *a* and *b* are constants for a given variety but can vary among varieties. From a physiological point of view, these two parameters may reflect the thermal requirement specificities for berry sugar accumulation both depending on grape variety and local climatic characteristics [72]. Considering the distinct climate characteristics between Europe and China, the parameters of *a* and *b* were re-estimated based on the observed harvest dates and sugar concentrations in China (Fig. 8).

### Assessment for freezing injury risks before harvest

To evaluate the suitability of each variety in a given region, the potential risk of experiencing freezing injury before harvest was evaluated. This was done by comparing the days (DOY) of initial occurrence when the temperature drops below 0°C in fall (or FFD) with the predicted GHDs for a specific target sugar concentration. We examined various factors of freezing injury risks, such as the FID, FIF and corresponding degree of freezing injury, which can cause damage from the FFD to the harvest in different grape-growing regions. To provide a holistic view of freezing injury, a CFI was developed by integrating the frequency, duration and severity of the freezing risk. To determine the freezing injury before harvest, a threshold temperature was established, and freezing injury occurred when daily minimum air temperature reached or fell below the threshold temperature. Therefore, the degree of freezing injury was divided into three categories: low (−2°C < *T*_min_ ≤ 0°C), medium (−4°C < *T*_min_ ≤ −2°C) and high (*T*_min_ ≤ −4°C). The weighted value of frequency (FP) and days (FD) for freezing injury increased progressively from low to high risk, with a ratio of 1:2:3, respectively [59, 73]. To comprehensively assess the low-temperature risk of winegrapes in different regions, FP and FD were normalized to eliminate the influences of dimensions. Finally, the evaluation of low-temperature risk was conducted by averaging the weights of the two indicators (Eqs 10–14).


(10) \begin{equation*} F{p}_n={F}_n/Y\times 100\% \end{equation*}

(11) \begin{equation*} FD=\sum \limits_{n=1}^3F{d}_n\times{W}_n \end{equation*}

(12) \begin{equation*} FP=\sum \limits_{n=1}^3F{p}_n\times{W}_n \end{equation*}

(13) \begin{equation*} {f}_i=\frac{F_i-{F}_{\min, i}}{F_{\max, i}-{F}_{\min, i}} \end{equation*}

(14) \begin{equation*} S=\frac{1}{2}\left({f}_1+{f}_2\right) \end{equation*}

where *F_n_* represents the number of years that experienced freezing injury of grapevine before harvest; *Y* is the number of total years with the value of 60 (1961–2020). *Fp_n_* and *Fd_n_* are the frequency and days of freezing injury, respectively; *W_n_* are the weighted values of the low, medium and high risks with values of 17, 33 and 50%, respectively; *S* is the risk of CFI for winegrape; *f_i_* is the normalized value. When *i* equals 1, *F_i_*, *F*_max*, i*_ and *F*_min*, i*_ are the original, maximum and minimum value of *FP;* when *i* equals 2, *F_i_*, *F*_max*, i*_ and *F*_min*, i*_ are the original, maximum and minimum value of *FD.*

### Statistical criteria and analysis

All data analysis, parameter estimation, statistical analysis and plotting were carried out with the R language. Three statistical indices were used to compare the simulated GHDs under different sugar concentrations with the corresponding observed values, including root mean square error (RMSE; Eq. 15), normalized root mean square error (NRMSE; Eq. 16) and the determination coefficient (*R*^2^; Eq. 17).


(15) \begin{equation*} RMSE=\sqrt{\frac{1}{n}{\sum}_{i=1}^n{\left({Y}_i-{X}_i\right)}^2} \end{equation*}

(16) \begin{equation*} NMSE=\frac{\sqrt{\frac{1}{n}{\sum}_{i=1}^n{\left({Y}_i-{X}_i\right)}^2}}{\overline{X}} \end{equation*}

(17) \begin{align*} {R}^2=\frac{\sum_{i=1}^n{\big({Y}_i-\overline{Y}\big)}^2}{\sum_{i=1}^n{\big({X}_i-\overline{X}\big)}^2} \end{align*}

where *X_i_* and *Y_i_* are the paired observed and simulated values, respectively. *^−^ X* and *^−^ Y* are the average values of observed and simulated variables, respectively. *n* is the number of observations.

## Supplementary Material

Web_Material_uhaf176

## Data Availability

The data underlying this article are available in the article and in its online supplementary material.
