# Supplementary material for: Warming temperature reduces the risk of pre-harvest freezing injury and modifies variety suitability in the main winegrape-growing regions of China
Source: Hortic Res. 2025 Jul 7;12(10):uhaf176. doi: 10.1093/hr/uhaf176 (PMC12532769; doi:10.1093/hr/uhaf176)
Supplement: Web_Material_uhaf176 [file web_material_uhaf176.zip › Supplementary-revised.docx]

**Table S1.** Abrupt year and periods before and after the abrupt climate change in the six wine producing regions.

| **Region** | **Station** | **Abrupt year** | **Period before the**  **abrupt climate change (P1)** | **Period after the**  **abrupt climate change (P2)** |
| --- | --- | --- | --- | --- |
| R1 | HM | 2001 | 1961—2000 (40) ^a^ | 2001—2020 (20) |
|  | YQ | 1991 | 1961—1990 (30) | 1991—2020 (30) |
| R2 | YC | 2002 | 1961—2001 (41) | 2002—2020 (19) |
|  | HN | 1998 | 1961—1997 (37) | 1998—2020 (23) |
| R3 | WW | 1998 | 1961—1997 (37) | 1998—2020 (23) |
|  | ZY | 1997 | 1961—1996 (36) | 1997—2020 (24) |
| R4 | LK | 1994 | 1961—1993 (33) | 1994—2020 (27) |
|  | PD | 1997 | 1961—1996 (36) | 1997—2020 (24) |
| R5 | HL | 1988 | 1961—1987 (27) | 1988—2020 (33) |
|  | MY | 1997 | 1961—1996 (36) | 1997—2020 (24) |
| R6 | TG | 1994 | 1961—1993 (33) | 1994—2020 (27) |
|  | LF | 2001 | 1961—2000 (40) | 2001—2020 (20) |

Note: ^a^ The number of years for each period was indicated in the parenthesis. HM, YQ, HN, YC, WW, ZY, LK, PD, HL, MY, TG, and LF represent Hami, Yanqi, Huinong, Yinchuan, Wuwei, Zhangye, Longkou, Pingdu, Huailai, Miyun, Linfen, and Taigu, respectively. R1, R2, R3, R4, R5, and R6 represent Xinjiang, Ningxia, Gansu, Shandong, Jing-Jin-Ji, and Shanxi regions, respectively.

**Table S2.** Observed 127 harvest dates (GHDs) and corresponding sugar concentrations for six different grape cultivars during 1994-2018 at eleven stations.

| **Cultivar** | **Region** | **Range values (average) of grape harvest date (d)** | **Range values (average) of sugar concentration (g/L)** | **References** |
| --- | --- | --- | --- | --- |
| Chardonnay | R2, R4 | 240 ~ 270 (253) | 165 ~ 220 (197) | 1, 7, 19 |
| Riesling | R1, R5 | 223 ~ 257 (240) | 160 ~ 220 (198) | 1, 7 |
| Pinot noir | R1, R2, R3 | 234 ~ 267 (245) | 179 ~ 220 (200) | 6, 11 |
| Merlot | R1, R3, R5 | 220 ~ 274 (254) | 170 ~ 227 (205) | 1, 2, 3, 4, 5, 6 |
| Syrah | R1, R5 | 224 ~ 265 (242) | 162 ~ 207 (189) | 1, 2, 3, 4, 5, 16 |
| Cabernet- Sauvignon | R1, R2, R3, R5 | 224 ~ 286 (260) | 160~ 230 (191) | 1, 3, 4, 6, 12, 14, 15, 17 |

Note: R1, R2, R3, R4, R5, and R6 represent Xinjiang, Ningxia, Gansu, Shandong, Jing-Jin-Ji, and Shanxi regions, respectively.

**References**

1. Ai L, Zhang Z. The research on the quality of main wine grapes in Shacheng region. *Acta Agriculturae Boreali-occidentalis Sinica* 2011;20(08):116-120
2. Bai Y. Three wine varieties of *Vitis vinifera* are suitable for cultivation in the Hexi corridor. *Journal of Gansu Forestry Science and Technolgy* 2021;46(02):43-45
3. Chang Y. Study on Maturity Index System of Winegrape in Tuha Region of Xinjiang. Yanglin: Northwest A&F University, Yangling, Shaanxi, China, 2019 (in Chinese).
4. Chen H. Comparative Study of Five Wine Grape Varieties in Changli, Hebei Province. Yanglin: Northwest A&F University, Yangling, Shaanxi, China, 2010 (in Chinese).
5. Ma Q, Ming X. Determination of grape maturation degree in Qilian grape manor of Hexi Corridor. *Sino-Overseas Grapevine & Wine* 2009;(03):50-52
6. Peng Y, Gao Z, Dong K *et al.* Effects of the suitable harvest time for main wine grapes and wine quality in Xinjiang Yanqi Basin. *Xinjiang Agricultural Sciences* 2018;55(07):1252-1263
7. Peng Y, Zhou L, Dong K *et al.* Suitable harvest periods for major white grape varieties in Xinjiang Province, China. *Food Industry Science and Technology* 2018;39(20):41-46
8. Shi Y, Qi Y, Zhang J *et al.* Study on the suitable harvest period of Cabernet Sauvignon grapes. *Hebei Fruit* 2007;(06):10-11
9. Song Y, Ta Y, Wang B. Studies on winegrape maturity and wine quality in Shihezi Zones. *Sino-Overseas Grapevine & Wine* 2005;(03):24-27
10. Su P, Yuan C, Yang L *et al.* Effect of different harvest dates on the quality of Pinot Noir grape and the resulting wine. *Modern Food Science and Technology* 2016;32(05):234-240
11. Tang Z, Chen S, Zhang X *et al*. Differences in the quality characteristics of grape and wine of different clones of newly introduced ‘Pinot Noir’ in Hexi Corridor. *China Brewing* 2021; 40(07):107-111
12. Tu T, Meng J, Wei X *et al*. Optimal harvest time of Cabernet Sauvignon grape in Xiangning, Shanxi. *Journal of Northwest A & F University (Natural Science Edition)* 2017;45(5):139-146
13. Wang Y, Lan Y, Sun L *et al*. Effect of extended harvest on the quality of Merlot grapes. *Acta Agriculturae Boreali-occidentalis Sinica* 2015;24(7):84-91
14. Zhang M, Dai H. The quality monitoring of Cabernet Sauvignon grape during maturity in the eastern foot area of Helan Mountain for the year 2015. *Journal of Agricultural Sciences* 2016;37(02):29-33
15. Zhang X, Kang Y, Yuan H *et al*. The quality evaluation of wine grape and its respond to weather condition. *Acta Ecologica Sinica* 2007;27(2):740-745
16. Zhang Y, Du Z, Yao Y *et al*. Quality changes and optimal harvesting period prediction of Syrah grapes in three production areas in Xinjiang Province. *China Brewing* 2019;38(11):80-84
17. Zhang Y, Yao Y, Du L *et al*. Quality analysis and optimal harvesting period of Cabernet Sauvignon grapes in multiple locations in Gansu Province based on principal component analysis. *Food Industry Science and Technology* 2020;41(02):227-232
18. Zhao Z, Zhang Z. Study on the main wine grape varieties of Pingdu aidier winery in Shandong Province. *Northern Horticulture* 2013;(13):15-18
19. Zhou P. Study on ecological regionalization of grape varieties in Beijing. Urumqi: Xinjiang Agricultural University. Urumqi, Xinjiang, China, 2004 (in Chinese).

**Table S3.** *F*_s-target_* values for the six grape cultivars as determined by the original GSR model (Parker et al., 2020) under different sugar concentrations.

| **Type** | **Cultivar** | **Sugar concentration**  **170 g/L (S170)** | **Sugar concentration**  **180 g/L (S180)** | **Sugar concentration**  **190 g/L**  **(S190)** | **Sugar concentration**  **200 g/L**  **(S200)** | **Sugar concentration**  **210 g/L**  **(S210)** |
| --- | --- | --- | --- | --- | --- | --- |
| White cultivar | Chardonnay (CH) | 2723 | 2772 | 2813 | 2892 |  |
|  | Riesling (R) | 2893 | 3002 | 2069 | 3225 |  |
| Red cultivar | Pinot noir (PN) |  | 2734 | 2788 | 2838 | 2899 |
|  | Merlot (M) |  | 2732 | 2794 | 2856 | 2904 |
|  | Syrah (S) |  | 2853 | 2934 | 2965 | 2987 |
|  | Cabernet- Sauvignon (CS) |  | 2865 | 2926 | 3031 | 3055 |

**Table S4.** Day of year (DOY) of above 10℃ for each winegrape-growing region in China.

| **Region** | **Station** | **Day of year (DOY) before the abrupt climate change (d)** | **Day of year (DOY) after the abrupt climate change (d)** |
| --- | --- | --- | --- |
| R1 | HM | 76~116 (98) ^a^ | 75~114 (95) |
|  | YQ | 89~116 (101) | 80~115 (98) |
| R2 | YC | 87~119 (105) | 80~113 (99) |
|  | HN | 90~124 (108) | 81~117 (100) |
| R3 | WW | 94~135 (113) | 87~130 (105) |
|  | ZY | 94~136 (114) | 91~129 (106) |
| R4 | LK | 91~119 (105) | 79~117 (96) |
|  | PD | 87~119 (98) | 72~111 (93) |
| R5 | HL | 93~125 (104) | 79~118 (100) |
|  | MY | 85~109 (96) | 79~117 (95) |
| R6 | TG | 87~141 (104) | 80~117 (98) |
|  | LF | 70~109 (89) | 61~104 (80) |

Note: ^a^ The average value of DOY was indicated in the parentheses. HM, YQ, HN, YC, WW, ZY, LK, PD, HL, MY, TG, and LF represent Hami, Yanqi, Huinong, Yinchuan, Wuwei, Zhangye, Longkou, Pingdu, Huailai, Miyun, Linfen, and Taigu, respectively. R1, R2, R3, R4, R5, and R6 represent Xinjiang, Ningxia, Gansu, Shandong, Jing-Jin-Ji, and Shanxi regions, respectively.

**Table S5.** Number and percentage of years that do not meet the required heat for grape maturity for the six grape cultivars during the P1 and P2 periods based on the improved GSR model.

| **Cultivar** | **Period** | **Sugar** | **R2** | **R3** | | **R5** | **R6** |
| --- | --- | --- | --- | --- | --- | --- | --- |
|  |  |  | **HN** | **WW** | **ZY** | **HL** | **TG** |
| CH | P1 | S170 | - | 2 (5.6%) | 2 (5.7%) | - | 1 (3.1%) |
|  |  | S180 | - | 2 (5.6%) | 3 (8.6%) | - | 1 (3.1%) |
|  |  | S190 | - | 4 (11.1%) | 5 (14.3%) | - | 1 (3.1%) |
|  |  | S200 | - | 8 (22.2%) | 10 (28.6%) | - | 1 (3.1%) |
|  | P2 | S170 | - | - | - | - | - |
|  |  | S180 | - | - | - | - | - |
|  |  | S190 | - | - | - | - | - |
|  |  | S200 | - | - | 1 (2.9%) | - | - |
| R | P1 | S170 | - | 3 (8.3%) | 4 (11.4%) | - | - |
|  |  | S180 | - | 8 (22.2%) | 10 (28.6%) | - | 1 (3.1%) |
|  |  | S190 | 1 (2.8%) | 15 (41.7%) | 18 (51.4%) | - | 1 (3.1%) |
|  |  | S200 | 2 (5.6%) | 26 (72.2%) | 28 (80.0%) | - | 1 (3.1%) |
|  | P2 | S170 | - | - | - | - | - |
|  |  | S180 | - | - | 1 (2.9%) | - | - |
|  |  | S190 | - | - | 1 (2.9%) | - | - |
|  |  | S200 | - | 1 (2.8%) | 2 (5.7%) | - | - |
| PN | P1 | S180 | - | - | - | - | - |
|  |  | S190 | - | - | - | - | - |
|  |  | S200 | - | 1 (2.8%) | 1 (2.9%) | - | - |
|  |  | S210 | - | 2 (5.6%) | 2 (5.7%) | - | - |
|  | P2 | S180 | - | - | - | - | - |
|  |  | S190 | - | - | - | - | - |
|  |  | S200 | - | - | - | - | - |
|  |  | S210 | - | - | - | - | - |
| M | P1 | S180 | - | 2 (5.6%) | 2 (5.7%) | - | - |
|  |  | S190 | - | 3 (8.3%) | 4 (11.4%) | - | - |
|  |  | S200 | - | 7 (19.4%) | 8 (22.9%) | - | - |
|  |  | S210 | - | 10 (28.6%) | 13 (37.1%) | - | - |
|  | P2 | S180 | - | - | - | - | - |
|  |  | S190 | - | - | - | - | - |
|  |  | S200 | - | - | 1 (2.9%) | - | - |
|  |  | S210 | - | - | 1 (2.9%) | - | - |
| S | P1 | S180 | - | 4 (11.1%) | 6 (17.1%) | - | - |
|  |  | S190 | - | 8 (22.2%) | 11 (31.4%) | - | 1 (3.1%) |
|  |  | S200 | 1 (2.8%) | 13 (36.1%) | 17 (48.6%) | - | 1 (3.1%) |
|  |  | S210 | 1 (2.8%) | 19 (52.7%) | 24 (68.6%) | - | 1 (3.1%) |
|  | P2 | S180 | - | - | - | - | - |
|  |  | S190 | - | - | 1 (2.9%) | - | - |
|  |  | S200 | - | - | 1 (2.9%) | - | - |
|  |  | S210 | - | 1 (2.7%) | 2 (5.7%) | - | - |
| CS | P1 | S180 | 1 (2.8%) | 15 (41.6%) | 17 (48.9%) | - | 1 (3.1%) |
|  |  | S190 | 1 (2.8%) | 23 (63.9%) | 26 (74.3%) | - | 1 (3.1%) |
|  |  | S200 | 3 (8.3%) | 29 (80.6%) | 30 (85.7%) | 1 (3.8%) | 1 (3.1%) |
|  |  | S210 | 6 (16.7%) | 32 (88.9%) | 35 (100%) | 1 (3.8%) | 1 (3.1%) |
|  | P2 | S180 | - | - | - | - | - |
|  |  | S190 | - | 1 (2.8%) | 2 (5.7%) | - | - |
|  |  | S200 | - | 1 (2.8%) | 2 (5.7%) | - | - |
|  |  | S210 | - | 5 (13.9%) | 6 (17.1%) | - | - |

Note: - represents zero. CH, CS, M, PN, R, and S represent Chardonnay, Cabernet-Sauvignon, Merlot, Pinot noir, Riesling, and Syrah, respectively. P1 and P2 represent the periods of before and after abrupt climate change, respectively.


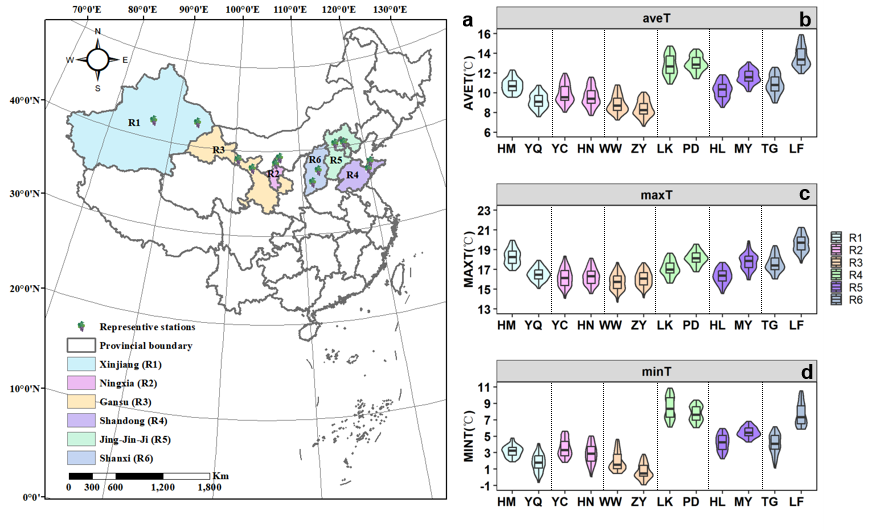


**Figure S1.** Main northern winegrape-growing regions of China and the 12 representative stations **(a)**, as well as their annual average **(b)**, maximum **(c)** and minimum **(d)** temperatures during 1961-2020 in each region.

Note: aveT, maxT, and minT represent annual average, maximum and minimum temperatures, respectively. HM, YQ, HN, YC, WW, ZY, LK, PD, HL, MY, TG, and LF represent Hami, Yanqi, Huinong, Yinchuan, Wuwei, Zhangye, Longkou, Pingdu, Huailai, Miyun, Taigu, and Linfen, respectively. R1, R2, R3, R4, R5, and R6 represent Xinjiang, Ningxia, Gansu, Shandong, Jing-Jin-Ji, and Shanxi regions, respectively.


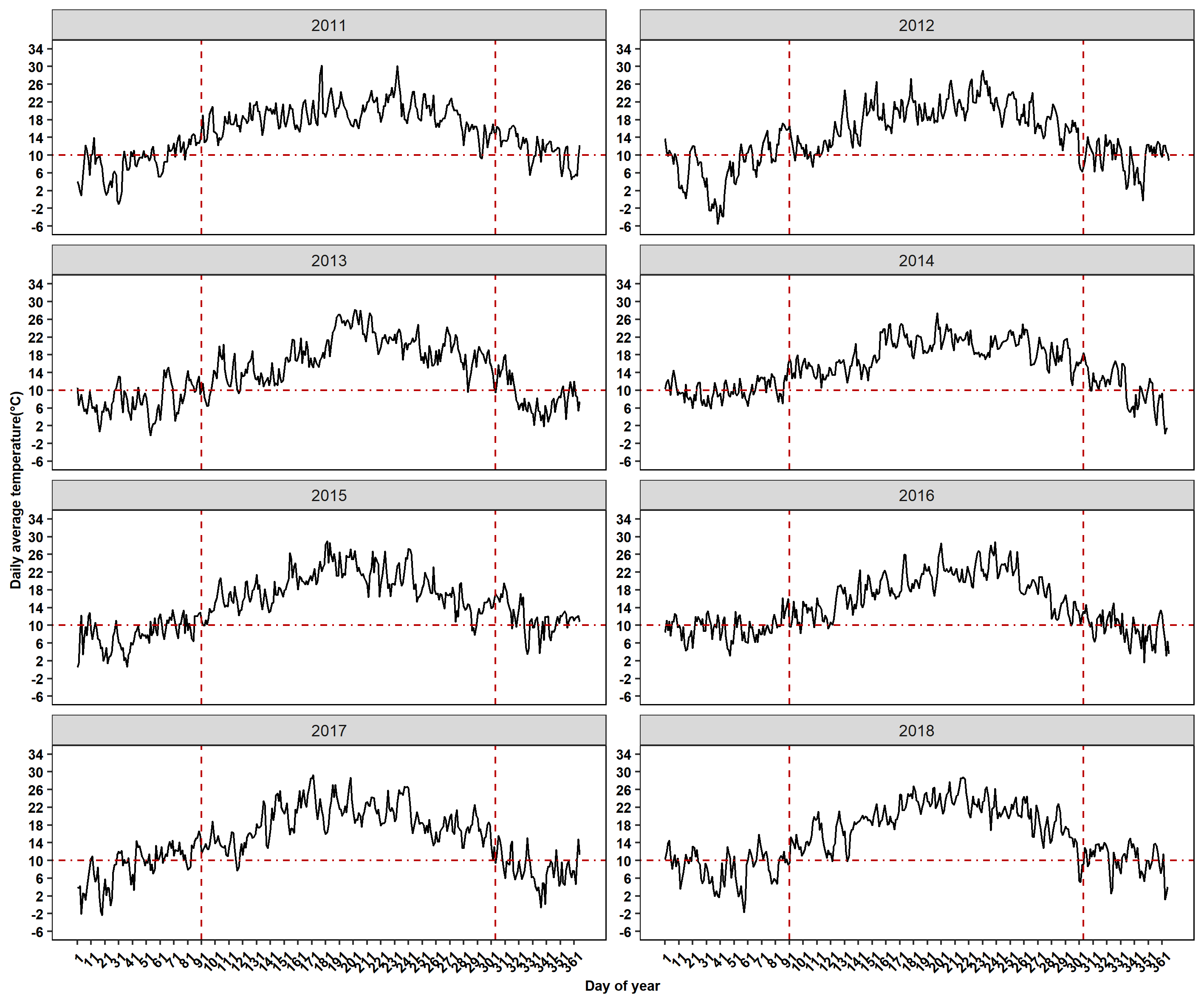


**Figure S2.** Daily average temperature in 2011-2018 at Bordeaux. The data stemmed from Yang et al., 2023.
